# HybFusion: A holistic Android malware detection framework with advanced feature fusion and ensemble learning

**DOI:** 10.1371/journal.pone.0349589

**Published:** 2026-06-12

**Authors:** Vu Minh Manh, Cho Do Xuan, Nguyen Thi Khanh Van

**Affiliations:** 1 Faculty of Information Security, Posts and Telecommunications Institute of Technology, Hanoi, Vietnam; 2 Lab Blockchain, Faculty of Information Security, Posts and Telecommunications Institute of Technology, Hanoi, Vietnam; 3 Faculty of Information Technology, Posts and Telecommunications Institute of Technology, Hanoi, Vietnam; DAV University, INDIA

## Abstract

Android malware detection remains a critical challenge due to the rapid increase in malware variants and the growing sophistication of obfuscation techniques. To address these issues, this paper introduces HybFusion, a holistic Android malware detection framework that integrates advanced feature fusion with ensemble learning to enhance detection effectiveness and reduce false positives. HybFusion combines two complementary feature types to overcome the limitations of existing methods in capturing a comprehensive representation of malware behaviors and semantics: (1) behavioral features extracted from the function call graph and embedded using the Graph Isomorphism Network, and (2) semantic permission features obtained from the *AndroidManifest.xml* file by applying a normalization process to convert permission identifiers into a permission sequence, which is then embedded using a lightweight pre-trained Transformer-based language model. This strategy enables better leveraging of semantic relationships among permissions while maintaining low computational cost. In addition, HybFusion adopts a stacking-based ensemble learning strategy that leverages the strengths of multiple classifiers to further improve detection robustness. Extensive experimental results demonstrate that HybFusion outperforms existing approaches across all evaluation metrics, achieving a recall of 99.24% and an F1-score of 99.27%.

## 1. Introduction

### 1.1. The problem of Android malware detection

The Android operating system is currently the most widely adopted platform for mobile devices, accounting for over 70% of the global mobile operating system market share [1]. However, this popularity has made Android a prime target for malware attacks. Android malware refers to malicious applications packaged in APK format. These are commonly distributed through the Google Play Store, third-party app stores, or via SMS, instant messaging services, and emails containing malicious links.

According to a report by Kaspersky, approximately 33.3 million mobile malware attacks were detected in 2024, with the number of banking Trojans increasing by 196% compared to the previous year [2]. Additionally, the Q3/2024 report from Gen Digital Inc. noted a 60% rise in banking malware and a 166% increase in spyware compared to the previous quarter [3]. Once infected, malware can steal sensitive data, gain unauthorized control over the device, and cause significant financial damage to users. Traditional detection approaches have become increasingly ineffective due to the growing volume and complexity of Android malware. This situation requires developing more advanced techniques to enhance malware detection performance.

### 1.2. Limitations of existing detection methods

Many methods have been proposed for Android malware detection, including signature-based techniques [4,5] and machine learning-based approaches [6–11]. Recently, machine learning-based methods have become a promising solution because of their ability to learn complex patterns from data, which helps detect previously unseen malware effectively.

For machine learning-based methods, the features extracted from data play a critical role in determining malware detection performance. There are three main categories of features commonly extracted from Android applications: static features [10–15], dynamic features [16–18], and hybrid features [19,20]. However, collecting dynamic and hybrid features typically requires building a complex analysis environment and is highly time-consuming. Therefore, most recent studies focus on static features because they are effective in detecting malware and require less time and resources, making them suitable for large-scale malware detection.

Static features can be categorized into two main groups: non-graph-based features [12,13,21–23] and graph-based features [7–10,15,24]. Among these, the function call graph (FCG) is a representative type of graph-based feature that has been widely adopted in recent studies [6–10,24,25]. An FCG is a directed graph where nodes represent functions within an application, and edges denote the calling relationships between them. This representation effectively models inter-function relationships and provides critical information about program behavior, thereby enhancing the effectiveness of malware detection models. However, based on a survey and analysis of existing research, we have identified several limitations in current FCG-based malware detection approaches, as outlined below:

(1) **Lack of comprehensive malware feature representation:** Recent studies on Android malware detection often rely on a single type of feature, either focusing on graph-based features such as the FCG [6–10,24–26], or relying solely on non-graph-based features such as permissions [12,21,27,28]. This approach leads to an incomplete representation of malware characteristics. In practice, an Android application consists of two primary components: the classes.dex file, which contains the executable code, and the *AndroidManifest.xml* file, which specifies configuration details and declares the permissions requested by the application. While malicious behaviors are reflected in the executable code, the permissions in the *AndroidManifest.xml* file provide crucial information about the resources and sensitive actions requested by the application. Relying on a single feature source makes the detection model less robust and more likely to miss critical malware indicators, especially when code obfuscation techniques are used to hide malicious behavior. Therefore, combining features extracted from both classes.dex and *AndroidManifest.xml* is essential to enhance representation capability and improve malware detection performance.
(2) **Limitations in permission feature extraction and representation:** Although permission features have been widely utilized in Android malware detection, the representation of permissions in existing methods still faces significant limitations. Specifically, traditional approaches typically encode the permission list as a binary vector, which is constructed based on a fixed permission dictionary or by selecting a subset of important permissions according to their frequency in the dataset [12,21,27,28]. However, these representations are discrete and fail to capture the actual usage context or the semantic relationships among permissions. Furthermore, relying on frequency-based selection limits the model to the characteristics of the specific training dataset and may result in missing infrequent yet highly discriminative permissions that indicate malicious behavior.
(3) **Limitations in malware classification methods:** Most existing studies focus on constructing feature representations of malware and then applying a single classifier to detect malicious samples [6–10,15]. However, this approach often suffers from limited detection performance, as the model entirely depends on a single algorithm for classification. This makes it susceptible to the inherent weaknesses of the chosen algorithm. Since each classification algorithm has its own strengths and weaknesses, relying on only one classifier fails to leverage the complementary advantages among different algorithms, thereby impacting the overall effectiveness of the malware detection model.

### 1.3. Proposed solutions

To address the above limitations, this study proposes a novel approach based on advanced feature extraction techniques from multiple components of Android applications, combined with ensemble learning. Specifically:

(1) To address the problem of **“Lack of comprehensive malware feature representation”**, we propose an advanced feature extraction method that combines both behavioral features from the classes.dex file and permission features from the *AndroidManifest.xml* file. Specifically, behavioral features are obtained by first decompiling classes.dex and constructing a FCG. Each node in the FCG is enriched with both graph structural features and semantic features derived from the function name, class name, and package name. The enriched FCG is then embedded using the Graph Isomorphism Network to produce a vector that represents the application’s behavior. Concurrently, all permissions requested by the application are extracted from the *AndroidManifest.xml* file and converted into a consistent textual representation through a normalization process. The normalized permissions are then concatenated into a permission sequence and embedded using the lightweight all-MiniLM-L6-v2 model to obtain a permission feature vector. These two vectors are then concatenated to form a comprehensive feature representation of the application. By combining features from multiple components of the APK file and applying advanced embedding techniques, the proposed method allows the model to construct a more complete representation of malware characteristics, thereby enhancing detection performance.
(2) To address the issue of **“Limitations in permission feature extraction and representation”**, we propose a method for extracting semantic features from the permission list of Android applications. Specifically, the list of permissions is extracted from the *AndroidManifest.xml* file and normalized into a consistent textual representation, which is then concatenated into a permission sequence. To capture semantic relationships among permissions, we utilize the all-MiniLM-L6-v2 model, a lightweight variant of SentenceTransformer, to map the sequence into a semantic embedding vector. This method allows the model to learn latent semantic information from the permission list, thereby providing a more comprehensive representation of permission usage contexts and inter-permission dependencies, which contributes to improving the effectiveness of malware detection.
(3) To address the issue of **“Limitations in malware classification methods”**, instead of relying on a single classifier, we propose adopting a stacking-based ensemble learning technique to combine the strengths of multiple classifiers, thereby enhancing the overall performance in the malware detection task.

### 1.4. Contributions of the paper

Some scientific contributions of this study include:

(1) A comprehensive Android malware detection model is proposed, integrating two essential types of features extracted from the main components of Android applications: behavioral features from the executable code (*classes.dex*) and permission features from the *AndroidManifest.xml* file. The behavioral features are representation-learned from the enriched FCG using the Graph Isomorphism Network, while the semantic permission features are obtained by normalizing the permission list into a permission sequence and embedding it using a pre-trained Transformer-based language model. This integration enables the construction of a more holistic behavioral profile and improves malware detection accuracy, especially when dealing with code obfuscation techniques.
(2) A semantic representation strategy for application permissions is proposed. Specifically, we introduce a normalization method that converts permission identifiers into a consistent textual form before encoding, making them more suitable for semantic embedding. The normalized permissions are then embedded using a lightweight pre-trained language model, namely all-MiniLM-L6-v2, to capture semantic relationships among permissions. This approach maintains low computational cost and does not require additional fine-tuning.
(3) A stacking-based ensemble classification technique is adopted to combine the strengths of multiple classifiers, thereby optimizing detection performance. This ensemble strategy improves both the accuracy and generalization capability of the proposed malware detection model.

## 2. Related works

### 2.1. Static analysis methods

Static analysis-based methods extract features directly from the content of APK files without requiring execution on a device or within a sandbox environment. To achieve this, these methods typically unpack the APK file into two main components: *AndroidManifest.xml* and *classes.dex*. The *AndroidManifest.xml* file contains configuration information such as application version, activities, required permissions, and other metadata, while the *classes.dex* file contains the application’s Dalvik bytecode. From these components, the extracted features can be categorized into two main groups: non-graph-based features and graph-based features.

#### 2.1.1. Non-graph-based features.

Regarding non-graph-based features, several commonly used features in Android malware detection models include permissions [12,21,27], API calls [13,29], intents [23], and image-based representations [14,22].

Permissions are an important and commonly used feature as they directly reflect the sensitive resources and functionalities that an application requests to access. This information is extracted from the *AndroidManifest.xml* file. The method in [12] selects 22 critical permissions out of a total of 135, achieving a detection accuracy of 93.62%. Reference [21] proposes the use of both native permissions and custom permissions for malware detection. After feature selection, 55 critical permissions were chosen as features, and the model achieved a detection accuracy of 96.95%. In study [27], a feature selection approach based on linear regression was proposed, reducing the feature set from 102 to 27 permissions, with an F1-score of 96.1% in malware detection. Methods relying solely on permission features result in limited detection effectiveness, as permissions can only capture coarse-grained features of the application.

Besides permission features, API calls also represent a critical feature for representing malicious behaviors, as applications must invoke APIs to interact with the Android operating system, thereby potentially indicating abnormal activities. Study [13] utilizes only critical API calls and their parameters to identify malicious behavior. Specifically, they performed frequency analysis to select APIs that were used more frequently in malware samples compared to benign. However, this method is limited when dealing with malware that employs less common or new APIs. Reference [29] proposes the combination of three feature types: API calls, permissions, and hardware features, where permissions and hardware features are extracted from *AndroidManifest.xml*, and API calls are derived from *classes.dex*. The authors introduce a Multi-Head Squeeze-and-Excitation Residual block architecture to mine and emphasize the intricate relationships between features from multiple perspectives, thereby enhancing malware detection performance, with the model achieving an accuracy of 96.48%.

Intents are also an important feature for identifying malicious behaviors in Android applications. They indicate how an application interacts with the system or other components, thereby helping to detect potential malicious activities. This feature is often combined with other features, such as permissions or API calls, to improve detection performance. The method in [23] combines intent features with permission features to detect Android malware. Experimental results show that this approach achieves a detection rate of 95.5%, significantly higher than the 91% obtained when using intent features alone.

Regarding image-based features, some methods convert the *classes.dex* file of applications into images, which can be in grayscale or RGB format. These images are then processed using Convolutional Neural Networks to automatically learn and extract features for malware detection tasks [14,22].

Recently, several studies have leveraged Transformer-based architectures to learn semantic representations of static features for Android malware detection. MalBERT [30] is a Transformer-based approach for Android malware detection. It extracts and preprocesses the AndroidManifest.xml file by removing non-informative terms, mainly repetitive words and punctuation. The processed text is then used to fine-tune a BERT model for semantic representation learning and malware classification. Experimental results show that the approach achieves an accuracy of 97.61%.

DetectBERT [31] is proposed to learn feature representations of Android application source code in the form of Smali. The method leverages DexBERT, a pretrained model based on the BERT architecture, to extract semantic features from disassembled Smali code at the class level. Since an application typically consists of a large number of Smali classes and DexBERT is limited to processing individual classes, DetectBERT adopts a correlated Multiple Instance Learning strategy to aggregate dependencies and interactions among class-level embeddings into an application-level representation for malware detection.

In addition, BERTroid [32] was proposed to learn semantic representations of permissions declared in the *AndroidManifest.xml* using a Transformer-based architecture. However, this approach primarily focuses on permission information at the application configuration level and does not incorporate behavioral features extracted from the executable code, which play a crucial role in modeling malicious behaviors.

In general, non-graph-based features can only capture discrete and coarse-grained characteristics of the application. These features lack the ability to represent contextual relationships or interactions between components in the source code. Therefore, they are not powerful enough to accurately reflect the overall behavior of the program. As a result, models that rely solely on these features may fail to detect sophisticated malware.

#### 2.1.2. Graph-based features.

Graph-based features can provide an effective abstract representation for modeling sequences of activities or relationships between components in Android applications. Various types of graph-based features have been proposed, including function call graphs, control flow graphs, and program dependence graphs. Among them, function call graphs have been widely adopted in recent studies due to their ability to capture the relationships between functions, thereby providing rich behavioral information and supporting effective malware detection.

The method in [24] is based on analyzing the centrality of sensitive API calls within the FCG of an application. In this approach, the FCG is considered a complex social network, and the centrality analysis of sensitive API calls is conducted to extract semantic features from the graph. Experimental results show that the method achieves a detection accuracy of 98%. However, it relies on a fixed list of sensitive APIs, which may become outdated as Android updates, potentially missing malicious behaviors that involve APIs not covered by the list.

A malware detection model based on the FCG is proposed in [25], where each node is represented by features related to opcode occurrence and API packages. Graph Neural Networks (GNNs) are then applied to extract higher-level features from the graph. Experimental results show that the model achieves a detection accuracy of 92.29%. Nevertheless, the node representation is limited, as it considers only the occurrence of opcodes and API packages as discrete features, while ignoring their semantic information.

The work in [8] proposes a method that prunes the FCG based on a predefined list of sensitive APIs. Each node in the pruned graph is then represented using semantic features of function names and triad attributes of the sensitive APIs. This approach has limitations, as relying on a fixed list of sensitive APIs for pruning may cause the model to overlook malicious behaviors that involve other APIs not included in the list.

In [7], the authors propose a malware detection method based on a sensitive function call graph. This graph is constructed by labeling sensitive nodes on the FCG, followed by extracting sensitive subgraphs and neighbor subgraphs to capture malicious behaviors from the application. Experimental results show that the model achieves an F1-score of 97.04%. However, this approach has certain limitations. First, it depends on a predefined list of sensitive APIs. In addition, it does not leverage node-level features, which could provide valuable information about the application’s behavior.

A feature enrichment method for nodes in the FCG based on Word2vec and node centrality is introduced in [9]. Specifically, if a node represents a system API, the API2vec model is employed to embed the corresponding package name. Conversely, for nodes representing user-defined functions, the Opcode2vec model is applied to embed the opcode sequences extracted from those functions. Additionally, the degree of each node is used as a weight for its feature vector. However, this approach has limitations, as it only exploits semantic features from package names, potentially overlooking critical information since functions within the same package may perform entirely different functions.

The work in [10] proposes a malware detection method based on the enriched FCG. The nodes in the FCG are augmented with features that combine graph-structured properties with semantic information related to function names, class names, and package names. This combination aims to enhance the modeling of application behavior at the function level. However, this method only exploits features from executable code and does not consider other important features, such as permissions, which are critical for identifying the sensitive resources or functionalities requested by the application.

Another approach in [11] constructs an API call graph by pruning the original FCG using a predefined list of sensitive APIs. The nodes in the API call graph are then represented using permission and opcode semantic features. However, this approach has several limitations. First, removing a large number of nodes from the original FCG may lead to the loss of important information, especially methods that are not directly linked to sensitive APIs but may still exhibit malicious behaviors. In addition, the effectiveness of this method strongly depends on the accuracy and completeness of the predefined sensitive API list.

Based on the review of the above studies, we compare static analysis-based Android malware detection methods in Table 1. The table summarizes these methods based on the extracted features, feature types, and publication year.

**Table 1:** Comparison of static analysis-based Android malware detection methods.

Study	Year	Features	Feature type
[12]	2018	Permissions	Non-graph
[27]	2021	Permissions	Non-graph
[21]	2021	Permissions	Non-graph
[13]	2013	API calls	Non-graph
[29]	2023	API calls, permissions, and hardware features	Non-graph
[23]	2017	Intents and permissions	Non-graph
[14]	2021	Grayscale images of binaries	Non-graph
[22]	2020	Color images of binaries	Non-graph
[30]	2021	Manifest metadata (BERT-based semantic features)	Non-graph
[31]	2024	Smali code	Non-graph
[32]	2024	Permissions	Non-graph
[24]	2019	Function call graph	Graph
[25]	2021	Function call graph	Graph
[7]	2022	Function call graph	Graph
[8]	2023	Function call graph	Graph
[10]	2024	Function call graph	Graph
[9]	2024	Function call graph	Graph
[11]	2024	API call graph	Graph

### 2.2. Dynamic analysis methods

In [16], the authors propose using system call sequences collected during application execution as input features for an LSTM network, achieving a recall of 96.6%. System call graphs are introduced in [17] as an important feature for malware detection. This method models system calls as graphs to capture structural dependencies among them. A Graph Convolutional Network is then applied to classify whether the system call graphs are malicious.

Given the limitations of system-call-based approaches under obfuscation, DroidCat is proposed in [33] to perform dynamic malware detection using method calls and inter-component communication intents as behavioral features. By avoiding reliance on system calls, permissions, and app resources, DroidCat demonstrates improved robustness compared to both static approaches and dynamic methods based on system calls.

Network activities are critical dynamic features commonly used in Android malware detection, as many malware samples perform unauthorized access or data theft through network connections. In particular, the work in [18] uses features from HTTP requests and TCP flows to distinguish between benign and malicious applications. Similarly, a set of seven features is extracted from network traffic in [34], including average packet size, average number of packets sent per flow, average number of packets received per flow, average number of bytes sent per flow, average number of bytes received per flow, ratio of incoming to outgoing bytes, and average number of bytes received per second. In addition, a deep learning-based framework is proposed in [35] for Android malware detection using network traffic features. Specifically, 86 network features are extracted from PCAP files using CICFlowMeter and fed into a CNN-LSTM model, where CNN captures local patterns in network flows, while LSTM models the sequential dependencies among features.

Dynamic analysis-based methods still face limitations when encountering malware that employs anti-emulation techniques to avoid triggering malicious behaviors, thereby reducing the effectiveness of feature collection. Additionally, some malicious behaviors may remain untriggered due to specific conditions, such as user interactions or timing, leading to the omission of critical malware features.

### 2.3. Hybrid analysis methods

Hybrid analysis combines static and dynamic analysis to leverage the advantages of both techniques, enabling a more comprehensive extraction of malware behaviors. However, since it integrates both approaches, this technique requires significant computational resources and processing time.

In [19], a hybrid model that combines static and dynamic analysis is proposed for Android malware detection using Tree Augmented Naive Bayes. Specifically, the method captures conditional dependencies between relevant static and dynamic features, including API calls, permissions, and system calls. The work in [20] extracts static features such as required permissions and sensitive API calls from the application’s source code, while dynamic features, including network activity, file system access, and interaction with the operating system, are collected during application execution. All these features are integrated and used as input to a machine learning classifier.

A hybrid approach in [36] combines dynamic features collected during application execution, such as system calls related to file operations and network activities, with static features including application permissions, intent actions, suspicious APIs, application components, and requested hardware resources. The resulting hybrid features are then used as input to an SVM classifier for Android malware detection.

The study in [37] introduces a CNN-Ensemble framework to combine static and dynamic features for Android malware detection. DEX files are first transformed into grayscale images and processed by a CNN to extract static features, while dynamic behaviors are captured via Monkey and Strace by monitoring system call frequencies. The extracted features are subsequently concatenated and input to a Gradient Boosting classifier for final classification.

Finally, the approach in [38] proposes a hybrid model, where static features are extracted by converting binary code into grayscale images and processing them with a CNN-LSTM, while dynamic features are collected from network traffic and reduced using Principal Component Analysis. The fused features are then classified via an ensemble voting mechanism.

Thus, it can be observed that existing Android malware detection approaches have not fully exploited features extracted from multiple components of the APK file. Most studies focus on a single source of information, such as executable code in *classes.dex* or metadata extracted from *AndroidManifest.xml*, which limits their ability to comprehensively model application behavior and makes them less robust against obfuscation techniques.

On the other hand, hybrid malware detection approaches that combine static and dynamic features, although capable of providing richer behavioral representations, often require high computational costs and face scalability challenges in practical deployment settings.

In this paper, we propose a holistic approach based entirely on static analysis, in which features extracted from multiple components of the APK file are systematically integrated. This approach aims to enhance the modeling of application behavior while maintaining efficiency and scalability for Android malware detection.

## 3. Proposed model

### 3.1. Architecture overview

**Fig 1:**
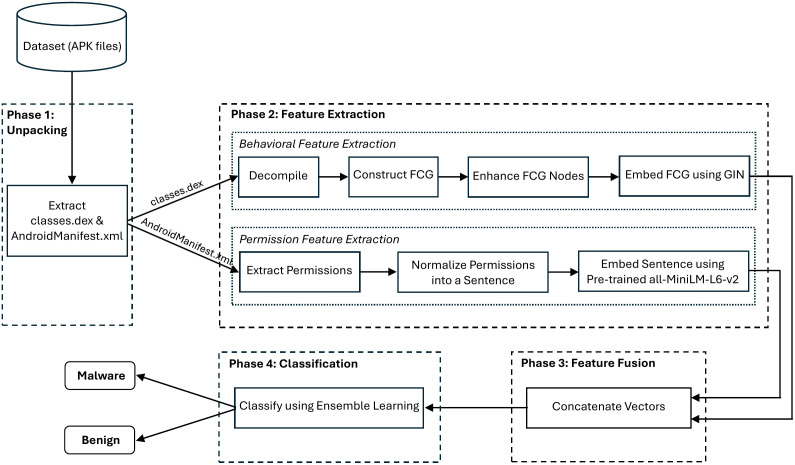
The overall architecture of the proposed HybFusion framework.

Fig 1 illustrates the overall architecture of the proposed HybFusion framework, consisting of four main phases.

(1) **Unpacking:** This phase unpacks the APK file to extract two key components: *classes.dex* and *AndroidManifest.xml*. The *classes.dex* file contains the application’s bytecode, which is utilized for analyzing behavioral features, while *AndroidManifest.xml* provides configuration information, including declared permissions, to support permission feature analysis.
(2) **Feature Extraction:** This phase extracts behavioral and permission features through two main sub-components:
  (a) ***Behavioral Feature Extraction:*** The *classes.dex* file is decompiled into Smali code and used to construct an FCG. Then, each node in the FCG is enriched by combining graph-structured features (in-degree, out-degree, closeness, Katz, and clustering coefficient) with semantic features of function names, class names, and package names, aiming to enhance the representational capability of the nodes. Finally, the enriched FCG is embedded using a Graph Isomorphism Network (GIN) to generate a behavioral feature vector for the application.
  (b) ***Permission Feature Extraction:*** From *AndroidManifest.xml*, permissions are extracted and normalized into a consistent textual representation. The normalized permissions are then concatenated into a permission sequence and embedded using the pre-trained all-MiniLM-L6-v2 model to obtain a permission feature vector.
(3) **Feature Fusion:** The behavioral feature vector and the permission feature vector are concatenated to create a fused feature vector. This vector integrates both behavioral and permission information and serves as the input to the classification stage.
(4) **Classification:** Based on the fused feature vector, HybFusion applies an ensemble learning technique to classify APK files as either malware or benign.

### 3.2. Feature extraction method

When analyzing an APK file, two main components are obtained: *classes.dex* and *AndroidManifest.xml*. The *classes.dex* file contains the bytecode and provides the primary source for analyzing and extracting behavioral features that reflect the application’s operations. Meanwhile, the *AndroidManifest.xml* file stores the list of permissions requested by the application and is used to analyze permission features. This section provides a detailed description of the feature extraction process from these two components, including behavioral features from the bytecode and permission features from the permission list.

#### 3.2.1. Behavioral feature extraction.

a) **Constructing the FCG**

To extract behavioral features from an Android application, the *classes.dex* file is first decompiled into Smali code, which is a human-readable intermediate format, using the Androguard tool [39]. Then, the entire Smali code is analyzed to construct the FCG.

**Definition 1.** A Function call graph is a directed graph, denoted as *FCG = (V, E)*, where *V* is the set of functions and *E* is the set of directed edges representing call relationships. Specifically, a directed edge from function *A* to function *B* indicates that *A* invokes *B* during the execution of the application.

The FCG plays an important role in modeling the structure and behavior of an application, as it provides detailed information about the relationships between functions and the program’s execution flow. However, in the original FCG, nodes only represent functions without incorporating detailed information about their roles or functionalities within the application. These details are important at the function level and can effectively reflect the application’s behavior. The lack of such information limits the capability of GNNs to learn meaningful representations from the graph. Therefore, to address this limitation and enhance the representational power of the FCG, the next step is to enrich the node information.

b) **Enhancing node representations in the FCG**

To represent nodes in the FCG, we adopt the node enrichment strategy from our previous work [10], which combines graph-structured properties with semantic information derived from package, class, and function names. For brevity, the detailed formulation is not repeated here.

In the context of FCGs, functions exhibit different roles and levels of influence within the execution flow. Functions with a high number of incoming or outgoing connections, or those located along important execution paths, often play a central role and are more likely to be involved in core application behaviors. Accordingly, structural features such as in-degree, out-degree, closeness centrality, Katz centrality, and clustering coefficient [40] are used to quantify the position and influence of each node, thereby capturing its role in the graph structure.

However, relying solely on structural information is insufficient to fully characterize the behavior of a function. Each node in the FCG corresponds to a specific function, and its functionality depends on its semantic context, including its class and package. These contextual elements provide important information about the function’s behavior, enabling the model to distinguish between functions that may share similar structural properties but exhibit different behaviors.

Therefore, integrating structural and semantic features enhances the representational capacity of the FCG. Each node v is represented by an initial feature vector $x_{v}$, obtained by combining its structural features and semantic representation.

c) **Embedding the enriched FCG using GIN**

Based on the enriched FCG, we propose using the GIN to extract behavioral features from the graph. GIN is a type of Graph Neural Network (GNN) introduced in [41], designed to improve the ability to distinguish between non-isomorphic graphs, which is an inherent limitation of many traditional GNN architectures. We choose GIN over other GNN models due to its advantage in effectively aggregating information from neighboring nodes and its strong discriminative power for complex graph structures. This is particularly important in Android malware detection, as malicious behaviors are often associated with specific structural patterns and abnormal interactions in the FCG.

GIN operates through a series of convolutional layers, where the feature vector of each node is updated by aggregating information from its neighboring nodes. At the *l*-th layer, the feature vector of node *v* is updated as follows:


$$\begin{array}{c} \overset{\left(l\right)}{\underset{v}{h}}={\text{MLP}}^{\left(l\right)}\left(\left(\text{1}+\epsilon^{\left(l\right)}\right)\cdot \overset{\left(l-\text{1}\right)}{\underset{v}{h}}+\sum_{u\in N(v)}\overset{\left(l-\text{1}\right)}{\underset{u}{h}}\right), \end{array}$$ (1)

where:

- $\overset{\left(l\right)}{\underset{v}{h}}$ is the feature vector of node *v* at the *l*-th layer, with $\overset{\left(\text{0}\right)}{\underset{v}{h}}=x_{v}$;
- $x_{v}$ is the initial feature vector of node *v*, obtained from the enriched node representation;
- N(v) denotes the set of neighboring nodes of node *v*;
- $\epsilon^{\left(l\right)}$ is a learnable parameter at the *l*-th layer;
- $\text{ML}{\text{P}}^{\left(\text{l}\right)}$ is a Multi-Layer Perceptron used in the *l*-th layer.

Intuitively, Equation (1) allows each node to refine its representation by combining its own previous features with the aggregated features of its neighboring nodes. Through this process, the model captures structural relationships and interaction patterns in the FCG. The MLP then applies a nonlinear transformation to the combined information, enabling the model to learn more expressive and discriminative node representations. Repeating this update across multiple layers allows the network to progressively capture higher-order structural dependencies in the graph.

To obtain the graph embedding vector, GIN employs a readout layer that aggregates the representations of all nodes at each layer and then concatenates the results from all layers. Specifically, the graph embedding vector, denoted as *h*_*G*_, is defined as follows:


$$\begin{array}{c} h_{G}=\text{Concat}\left(\sum_{v\in V}\overset{\left(l\right)}{\underset{v}{h}}\;|\;l=\text{0},\text{1},\ldots ,L\right), \end{array}$$ (2)

where *V* denotes the set of all nodes in the FCG, and *L* is the number of convolutional layers.

In this paper, we design the GIN architecture with two convolutional layers (*L = 2*) to balance computational efficiency and representation learning capability. After applying GIN, we obtain the output vector *h*_*G*_, which represents the behavioral characteristics of the application.

#### 3.2.2. Permission feature extraction.

Permission features play an important role in Android malware detection and have been widely adopted in previous studies. Typically, the list of permissions is extracted from the *AndroidManifest.xml* file and represented using one of two common approaches: (1) representing them as a binary vector based on a permission dictionary constructed from the entire dataset; or (2) selecting a subset of important permissions based on their frequency in both malware and benign samples, and then encoding each as a binary vector whose dimensions correspond to the selected permissions.

However, both of the above approaches have some notable limitations. First, such representations are discrete and fail to reflect the actual usage context of the permissions, which reduces the ability to distinguish between malicious and benign applications. For example, the RECORD_AUDIO permission may be requested by both legitimate calling apps and spyware, but with entirely different purposes. Second, these traditional representations overlook the semantic relationships among permissions, while many malicious behaviors can only be detected through the combination of several related permissions used together. For instance, the co-occurrence of READ_CONTACTS and INTERNET may indicate potential user data exfiltration.

To overcome these limitations, we propose a novel method that learns semantic features from the permission list using a pre-trained language model. This approach allows for effective extraction of latent semantic relationships between permissions.

a) **Permission extraction and normalization**

First, we extract all permissions of an APK file from the *AndroidManifest.xml* file. Subsequently, the permissions are normalized by removing prefixes and replacing underscores with spaces (e.g., ‘READ_CONTACTS’ is converted to ‘READ CONTACTS’). This step converts permission identifiers into a normalized textual form, making them more suitable for semantic embedding. The normalized permissions are then concatenated into a permission sequence and used as input to the embedding model.

b) **Permission embedding with all-MiniLM-L6-v2**

After normalization, the permission sequence of each application is used as input to a pre-trained language model to obtain a semantic vector. Specifically, we use the all-MiniLM-L6-v2 model [42], which is based on the SentenceTransformer architecture and is pre-trained to capture semantic relationships within a sequence [43].

The all-MiniLM-L6-v2 model is a lightweight version of MiniLM, consisting of 6 transformer layers, a 384-dimensional hidden size, and approximately 22 million parameters. With this architecture, the model is capable of encoding an input sequence into a 384-dimensional embedding vector while maintaining a good balance between performance and computational cost. We choose this model due to its compact size compared to larger language models, while still effectively capturing semantic relationships within a sequence. This makes it particularly suitable for large-scale data processing in Android malware detection.

For each permission sequence *p* of an application, we use the all-MiniLM-L6-v2 model to embed it into a semantic vector *e*_*p*_, which represents the permission feature of the application.

By normalizing permissions before encoding, the model can effectively capture semantic relationships among permissions while maintaining computational efficiency, without relying on large and complex models such as BERT.

### 3.3. Feature fusion method

The behavioral feature vector *h*_*G*_ and the permission feature vector *e*_*p*_ are concatenated horizontally to form a fused feature vector. This combined representation enables the model to simultaneously capture both the behavioral patterns from the application’s source code and the semantic relationships among the requested permissions. By integrating these two types of features, the model constructs a rich and comprehensive feature profile for each APK file, thereby enhancing its effectiveness in malware detection.

### 3.4. Classification

Based on the features extracted from APK files, traditional malware detection methods often rely on a single classifier to classify applications as either malware or benign. However, this approach is easily affected by the inherent limitations of individual algorithms, leading to unstable detection performance, especially when dealing with diverse and complex malware samples.

To address this limitation and enhance detection effectiveness, we propose using a stacking-based ensemble learning method. This technique combines multiple models to leverage the strengths of different classification algorithms [44]. In stacking, multiple base learners are trained on the same dataset to produce initial predictions, which are then used as input for a meta learner to make the final classification decision. This approach not only improves accuracy but also enhances generalization by combining the diversity of base models.

Specifically, in this paper, we design a two-level stacking architecture as follows:

- **Level 1 (Base learners):** Four independent classification models are selected as base learners, including K-Nearest Neighbors (KNN), Multi-Layer Perceptron (MLP), XGBoost, and Random Forest (RF). The diversity among these algorithms enables complementary strengths, helping to reduce overfitting and improve the model’s ability to detect complex patterns in the data. Each base learner is trained on the fused feature vector obtained from the feature fusion stage.
- **Level 2 (Meta learner):** LightGBM is chosen as the meta learner to aggregate the outputs of the base learners and make the final classification decision. LightGBM is selected due to its scalability, strong generalization capabilities, and high performance in binary classification tasks.

To reduce overfitting during the training of the meta learner, we adopt the k-fold cross-validation technique with *k = 5*. Specifically, the training data is divided into five non-overlapping subsets. In each fold, four subsets are used to train the base learners, while the remaining one is used to generate meta-features based on their predictions. This process is repeated five times to ensure that all training samples contribute to the creation of meta-features. The resulting meta-features are then combined into a meta dataset, which serves as the input to train the final classification model using LightGBM. This method not only helps reduce overfitting, but also enhances the stability and generalization capability of the model across different datasets.

## 4. Experiments and evaluation

### 4.1. Experimental dataset

The primary experimental dataset used in this study consists of 35,753 Android applications, including 17,813 malware samples and 17,940 benign samples. The APK files were collected from January 2018 to December 2023, covering multiple Android releases from API level 27 (Android 8.1) to API level 34 (Android 14), which enables the dataset to capture the evolution of Android permissions and APIs across different platform versions. The malware samples were collected from VirusShare [45], while the benign samples were sourced from AndroZoo [46]. To ensure label reliability, all samples were further verified using the VirusTotal API. Specifically, an APK file was labeled as benign if none of the antivirus engines in VirusTotal reported it as malicious, and labeled as malware if at least 10 engines identified it as malicious. Samples with inconsistent or ambiguous detection results were excluded from the dataset. Table 2 provides a detailed summary of the primary experimental dataset.

**Table 2:** Details of the primary experimental dataset.

Category	Source	Number of samples	Avg # nodes of FCG	Avg # edges of FCG
Malware	VirusShare	17,813	28,382.94	81,866.95
Benign	AndroZoo	17,940	5,938.94	18,476.87

In addition to the primary experimental dataset, the publicly available Drebin benchmark dataset [47] was used for external validation in the cross-dataset generalization experiment. The Drebin dataset contains 5,560 malware samples from 179 different malware families.

### 4.2. Evaluation criteria and scenarios

#### 4.2.1. Evaluation criteria.

To evaluate the performance of HybFusion, we employ four commonly used metrics, including Accuracy, Precision, Recall, and F1-score. They are defined as follows:


$$\begin{array}{c} \text{Accuracy}=\frac{TP+TN}{TP+TN+FP+FN}, \end{array}$$ (3)

$$\begin{array}{c} \text{Precision}=\frac{TP}{TP+FP}, \end{array}$$ (4)

$$\begin{array}{c} \text{Recall}=\frac{TP}{TP+FN}, \end{array}$$ (5)

$$\begin{array}{c} \text{F1}-\text{score}=\text{2}\times \frac{\text{Precision}\times \text{Recall}}{\text{Precision}+\text{Recall}}, \end{array}$$ (6)

where TP (True Positive) is defined as the number of malware applications correctly classified as malware, TN (True Negative) as the number of benign applications correctly classified as benign, FP (False Positive) as the number of benign applications incorrectly classified as malware, and FN (False Negative) as the number of malware applications incorrectly classified as benign.

Additionally, we use the confusion matrix to visually demonstrate the classification performance and make it easier to compare with other models. This matrix shows the values of TP, TN, FP, and FN, providing more detailed information about the model’s correct and incorrect predictions on the experimental dataset.

Classification performance was evaluated using 5-fold cross-validation, and the reported results correspond to the average values across the five folds.

#### 4.2.2. Evaluation scenarios.

To evaluate the effectiveness of the proposed HybFusion model, we design nine evaluation scenarios as described below:

- **Scenario 1:** This scenario evaluates the effectiveness of feature fusion across multiple APK components, integrating behavioral features extracted from *classes.dex* and permission features extracted from *AndroidManifest.xml*. The results are compared with single-feature models to quantify each feature type’s contribution and confirm the effectiveness of fusion.
- **Scenario 2:** This scenario assesses the effectiveness of permission feature extraction using the pre-trained language model all-MiniLM-L6-v2. The proposed approach is compared with traditional permission representation methods, including: (1) binary vector representation based on a predefined permission dictionary, and (2) representation based on a subset of important permissions selected using frequency statistics. In addition, we compare our approach with Word2vec, a widely used semantic embedding technique, to demonstrate the advantages of using the pre-trained MiniLM model for permission feature representation.
- **Scenario 3:** This scenario evaluates the effectiveness of the stacking-based ensemble learning technique by comparing the classification performance of the stacking architecture with individual classification algorithms, including KNN, MLP, XGBoost, and RF.
- **Scenario 4:** This scenario investigates the impact of key model parameters on detection performance. Specifically, we focus on two aspects: the number of GIN layers used for embedding the FCG, and the choice of meta learner in the stacking-based ensemble classifier.
- **Scenario 5:** This scenario compares the performance of HybFusion with other methods on the primary experimental dataset.
- **Scenario 6:** This scenario evaluates the cross-dataset generalization capability of HybFusion by comparing it with other methods on the Drebin benchmark dataset. All models are trained on the primary experimental dataset described in Section 4.1 and evaluated on unseen Drebin malware samples.
- **Scenario 7:** This scenario assesses the robustness of the proposed HybFusion model under adversarial evasion attacks, where four attack types are considered: Permission stuffing, FCG poisoning, Name obfuscation, and Permission reordering.
- **Scenario 8:** This scenario evaluates the robustness of HybFusion under common obfuscation techniques, including class and method renaming, call indirection, constant string encryption, and manifest randomization.
- **Scenario 9:** This scenario analyzes the computational cost of HybFusion in terms of runtime and resource consumption, including average processing time per sample, peak RAM usage, peak VRAM usage, and throughput measured in applications processed per hour.

Unless otherwise specified, Scenarios 1–5 and 7–9 are conducted on the primary experimental dataset described in Section 4.1.

### 4.3. Hyperparameter configuration

Table 3 summarizes the hyperparameter settings used in the proposed model. These hyperparameters were selected based on commonly adopted configurations in previous related studies and further refined through lightweight manual tuning on a validation set to ensure stable performance.

**Table 3:** Hyperparameter configuration of the proposed model.

Component	Hyperparameter	Value
All-MiniLM-L6-v2	Number of layers	6
	Output dimension	384
	Maximum sequence length	256
	Pooling	Mean pooling
GIN	The number of convolutional layers	2
	Optimizer	Adam
	Epochs	100
	Batch size	64
	Learning rate	0.005
	Activation function	ReLU
	Loss function	CrossEntropyLoss
Word2vec	Window	2
	Min_count	1
	Epochs	15
	Vector_size	100
KNN	N_neighbors	3
RF	N_estimators	100
	Random_state	42
	Max_depth	40
MLP	Hidden_layer_sizes	[64, 32]
	Activation	ReLU
	Solver	Adam
	Max_iter	500
	Random_state	42
XGBoost	N_estimators	100
	Learning_rate	0.1
	Max_depth	3
	Random_state	42
LightGBM	N_estimators	16
	Random_state	42
	Num_leaves	60

### 4.4. Experimental results

#### 4.4.1. Experimental results of scenario 1.

This scenario aims to evaluate the effectiveness of combining behavioral features and permission features compared to using each feature type individually. Table 4 presents the model’s performance under the three different settings.

**Table 4:** Comparison of model performance using individual and combined features (%).

Feature Type	Accuracy	Precision	Recall	F1-score
Permission feature only	97.51	98.18	96.80	97.49
Behavioral feature only	98.57	98.93	98.21	98.57
Fused feature	**99.27**	**99.30**	**99.24**	**99.27**

The results in Table 4 show that both types of features achieve high detection performance when used independently. Specifically, when using only permission features, the model achieves an accuracy of 97.51% and an F1-score of 97.49%. This demonstrates the effectiveness of our proposed semantic permission feature extraction method in capturing an application’s access privileges.

Compared to the permission features, the behavioral features demonstrate superior performance. When the model uses only behavioral features, it achieves an F1-score of 98.57%, which is 1.08% higher than when using only permission features. This indicates that function call graph-based behavioral features provide richer information about the application’s operational behavior, thereby improving the model’s ability to distinguish between benign and malicious applications.

Notably, when combining both feature types, the model achieves the highest performance across all metrics, with an accuracy of 99.27%, a precision of 99.30%, a recall of 99.24%, and an F1-score of 99.27%, outperforming the use of each feature type individually. This demonstrates that the two feature types are complementary, and their integration enables the model to capture more comprehensive information about the application, thereby significantly improving malware detection effectiveness.

**Fig 2:**
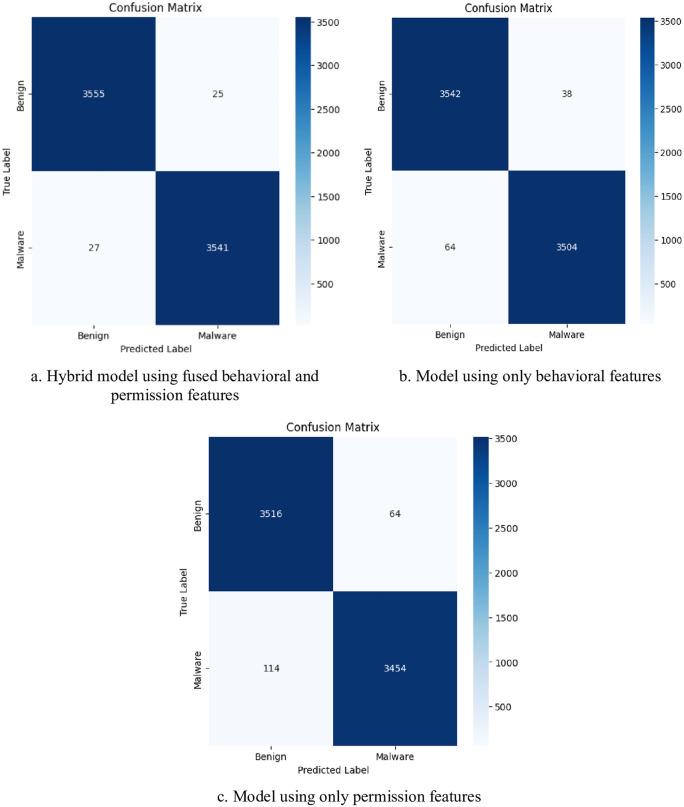
Confusion matrices under different feature configurations.

Fig 2 presents the confusion matrices of three models: (a) the proposed model using fused behavioral and permission features, (b) the model using only behavioral features, and (c) the model using only permission features. Specifically, Fig 2a shows that when both types of features are combined, the model correctly predicts 3,541 out of 3,568 malware samples, achieving a detection rate of 99.24%. Meanwhile, only 25 out of 3,580 benign samples are incorrectly predicted as malware, corresponding to a false prediction rate of just 0.76%. These results demonstrate the high accuracy and stable classification performance of the model. Compared to Fig 2b, when using only behavioral features, the model correctly predicts 3,504 malware samples (98.21%) and incorrectly predicts 64 samples. Fig 2c shows that the model using only permission features yields lower performance, correctly predicting 3,454 malware samples (96.80%) while incorrectly predicting 114 samples. These findings further confirm that each feature type, when processed and extracted using the proposed method, produces good results in malware detection. However, combining both feature types leads to superior performance.

#### 4.4.2. Experimental results of scenario 2.

This scenario aims to evaluate the effectiveness of the proposed permission semantic feature extraction method, which employs the all-MiniLM-L6-v2 language model, by comparing it with three other permission feature extraction approaches. Specifically:

(1) The permission list of each APK file is represented as a binary vector based on a pre-constructed permission dictionary.
(2) Based on the frequency of permissions in malicious and benign files, the 20 most significant permissions are selected using the approach outlined in [12]. Each APK file is subsequently represented by a 20-dimensional binary vector.
(3) The normalized permission list is embedded using the Word2vec technique to generate a feature vector.

It should be noted that this scenario focuses solely on assessing the quality of the permission feature vectors generated by each method, without combining them with any other features. These permission feature vectors are then used as input for a classifier to compare the representational effectiveness of each approach. Detailed experimental results are presented in Table 5.

**Table 5:** Classification performance of different permission feature extraction methods (%).

Method	Accuracy	Precision	Recall	F1-score
One-hot (permission dictionary)	96.28	97.52	94.96	96.22
Frequency-based	96.36	97.56	95.10	96.31
Word2vec embedding	96.73	96.67	96.78	96.72
Proposed method (all-MiniLM-L6-v2)	**97.51**	**98.18**	**96.80**	**97.49**

The results in Table 5 indicate that the proposed method using the all-MiniLM-L6-v2 model achieves the highest classification performance. Specifically, compared to the two traditional permission feature extraction methods based on a permission dictionary and frequency based approaches, our method improves accuracy by approximately 1.15% and F1-score by about 1.18%. These results demonstrate the superior capability of the all-MiniLM-L6-v2 model in learning semantic representations from normalized permission lists.

Notably, compared to Word2vec, a widely used semantic embedding technique, the all-MiniLM-L6-v2 method still achieves an F1-score 0.77% higher. This suggests that modern pre-trained language models like MiniLM can capture contextual information and relationships between permissions more effectively than traditional embedding methods.

**Fig 3:**
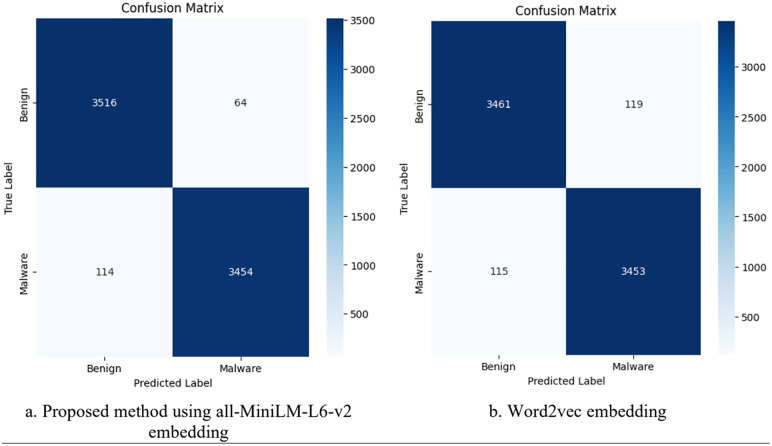
Comparison of confusion matrices between all-MiniLM-L6-v2 and Word2vec embeddings.

Fig 3 presents the confusion matrices of two permission list embedding methods: (a) the proposed method using the all-MiniLM-L6-v2 language model and (b) the method using Word2vec. It can be observed that the proposed method reduces the number of benign files incorrectly predicted as malware by approximately 46% (from 119 files to 64 files). This result confirms that lightweight pre trained language models like MiniLM can be effectively utilized to extract semantic features from normalized permission lists.

In summary, the results confirm that normalizing the permission list into a permission sequence and embedding it using a lightweight pre-trained language model such as all-MiniLM-L6-v2 can significantly enhance malware detection effectiveness. This approach enables better exploitation of latent semantic relationships among permissions, thereby increasing classification accuracy.

#### 4.4.3. Experimental results of scenario 3.

**Fig 4:**
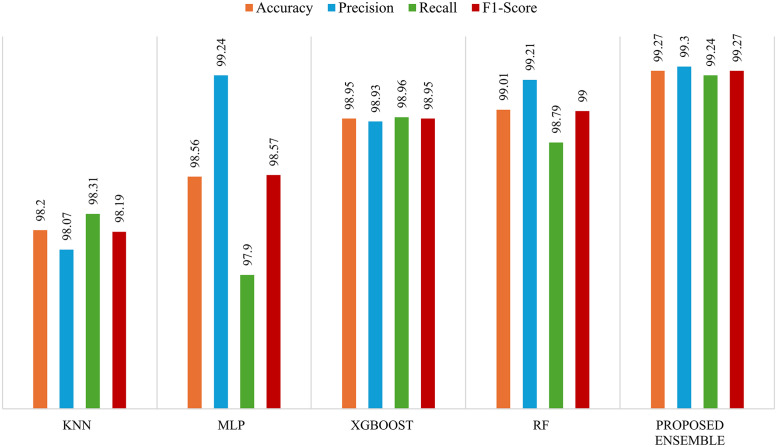
Performance comparison between individual classifiers and the stacking-based ensemble (%).

This scenario aims to evaluate the effectiveness of the classification model using the proposed stacking-based ensemble learning technique, compared to several popular individual classifiers, including KNN, MLP, RF, and XGBoost. Fig 4 presents a detailed summary of the experimental results for this scenario.

The results in Fig 4 indicate that the proposed model outperforms all individual classifiers across all evaluation metrics. Specifically, when comparing the highest and lowest values for each metric, the proposed model improves accuracy by 1.07%, precision by 1.23%, recall by 1.34%, and F1-score by 1.08%. These results demonstrate that the stacking-based ensemble learning technique effectively leverages the strengths of each base learner, thereby enhancing the overall performance of the malware detection model.

To further evaluate the stability and reliability of these results, Table 6 reports the mean and standard deviation obtained from 5-fold cross-validation. The proposed ensemble model achieves an F1-score of 99.27 ± 0.03, compared with 99.00 ± 0.08 for RF, which is the best-performing individual classifier. This shows that the proposed model not only provides higher classification performance, but also exhibits greater stability across multiple folds.

**Table 6:** Comparison of classifier performance across five folds with mean and standard deviation (%).

Classifier	Accuracy	Precision	Recall	F1-score
KNN	98.20 ± 0.07	98.07 ± 0.05	98.31 ± 0.09	98.19 ± 0.07
MLP	98.56 ± 0.04	99.24 ± 0.27	97.90 ± 0.19	98.57 ± 0.04
XGBoost	98.95 ± 0.04	98.93± 0.10	98.96 ± 0.05	98.95 ± 0.04
RF	99.01 ± 0.07	99.21 ± 0.07	98.79 ± 0.09	99.00 ± 0.08
Proposed Ensemble	**99.27** ± **0.03**	**99.30** ± **0.06**	**99.24** ± **0.07**	**99.27** ± **0.03**

Furthermore, the individual classifiers also achieve strong predictive performance, with F1-scores ranging from 98.19% (KNN) to 99.00% (RF). This further confirms that the proposed intelligent feature extraction and fusion strategy provides a rich representation of Android applications, thereby offering effective support for machine learning algorithms in classification tasks.

#### 4.4.4. Experimental results of scenario 4.

This scenario evaluates how variations in two key model parameters affect detection performance. These parameters include the number of GIN layers applied for graph embedding and the choice of meta learner used in the stacking-based ensemble classifier. Results are detailed in Tables 7 and 8.

**Table 7:** Performance of the proposed model with varying GIN layers (%).

GIN Layers	Accuracy	Precision	Recall	F1-score
1	99.09	98.91	99.27	99.09
2	**99.27**	**99.30**	**99.24**	**99.27**
3	99.19	99.24	99.13	99.19
4	99.15	99.24	99.05	99.14

**Table 8:** Performance of the proposed model with varying meta learner configurations (%).

Meta Learner	Accuracy	Precision	Recall	F1-score
XGBoost	99.23	99.49	98.96	99.23
Logistic Regression	99.24	99.27	99.22	99.24
LightGBM	**99.27**	**99.30**	**99.24**	**99.27**
SVM	99.19	99.19	99.19	99.19
CatBoost	99.20	99.27	99.13	99.20

Table 7 presents the performance metrics of the proposed model with varying numbers of GIN layers, from 1 to 4, while keeping all other parameters constant. The results indicate that the model achieves its best performance when using two GIN layers, reaching an accuracy of 99.27%, precision of 99.30%, recall of 99.24%, and F1-score of 99.27%. This suggests that two layers provide sufficient depth for the GIN to effectively learn expressive representations from the enriched FCG. With only one layer, the F1-score drops to 99.09%, implying that the network lacks sufficient depth to fully capture the structural patterns of the graph. As the number of layers increases to 3 and 4, the performance slightly declines, with F1-score of 99.19% and 99.14%, respectively. This decline likely reflects overfitting or the loss of relevant information due to excessive aggregation, suggesting that deeper layers may introduce noise and reduce the model’s ability to distinguish important graph patterns. These findings highlight that a two-layer GIN configuration offers the best trade-off between expressiveness and generalization for this task.

Table 8 presents the performance of the proposed model with different classifiers serving as the meta learner in the ensemble framework. Among the tested options, LightGBM yields the best results with an F1-score of 99.27%, followed by Logistic Regression (99.24%) and XGBoost (99.23%). Although SVM and CatBoost also achieve strong performance, their scores are slightly lower. Overall, these results demonstrate that the ensemble framework is robust across various meta learners, with gradient-boosting-based methods offering a marginal advantage in classification accuracy.

#### 4.4.5. Experimental results of scenario 5.

In this scenario, we compare HybFusion with state-of-the-art approaches on the primary experimental dataset. Specifically, the selected methods include DexRay [14], NATICUSdroid [21], Permission [28], Malscan [24], Mamadroid [48], and S^3^Feature [7]. These are well-known and open-source methods for Android malware detection. DexRay converts bytecode from DEX files into grey-scale images and uses them as the primary features for detection; NATICUSdroid leverages both native permissions and custom permissions to construct features; Permission applies a genetic algorithm to select an optimal subset of requested permissions; Malscan is a malware detection method based on analyzing the centrality of sensitive API calls within a function call graph of an application; Mamadroid proposes constructing abstracted API sequences from a function call graph, subsequently using a Markov model to transform these sequences into a feature vector representing the application; S^3^Feature extracts sensitive subgraphs and neighbor subgraphs from the sensitive function call graph to mine suspicious behaviors of applications. Detailed comparison results between the proposed model and the other methods are presented in Table 9.

**Table 9:** Performance comparison with other methods (%).

Method	Accuracy	Precision	Recall	F1-score
DexRay [14]	96.67	96.80	96.52	96.66
NATICUSdroid [21]	97.72	97.18	98.39	97.78
Permission [28]	97.36	96.12	98.72	97.40
Malscan [24]	97.82	97.58	98.07	97.82
Mamadroid [48]	66.30	60.40	96.10	73.70
S^3^Feature [7].	85.67	95.38	74.87	83.89
HybFusion	**99.27**	**99.30**	**99.24**	**99.27**

The results in Table 9 demonstrate that the proposed method outperforms all other methods across all evaluation metrics. Specifically, our model achieves an accuracy of 99.27%, precision of 99.30%, recall of 99.24%, and F1-score of 99.27%, clearly outperforming the next best methods, Malscan with an F1-score of 97.82% and NATICUSdroid with an F1-score of 97.78%. These findings highlight the proposed model’s accurate and stable malware detection capability.

Among the remaining methods, DexRay shows stable performance, achieving an accuracy of 96.67% and an F1-score of 96.66%, but still significantly lower than our approach. The main reason is that DexRay relies solely on grayscale images converted from bytecode, without deeply leveraging behavioral and permission features. NATICUSdroid utilizes both native and custom permissions, resulting in the highest recall among the compared methods at 98.39%, with an F1-score of 97.78%. However, its overall accuracy remains low due to the lack of behavioral features from the function call graph. The Permission method employs a genetic algorithm to optimize the permission set, achieving a recall of 98.72%, but its precision is notably lower at 96.12%, reflecting an imbalance in classification performance.

For graph-based methods, Malscan achieves an F1-score of 97.82%, which is still noticeably lower than our proposed model. This is primarily because the method does not leverage permission features or employ ensemble learning techniques. S^3^Feature shows low performance, with an accuracy of 85.67% and a recall of 74.87%. This is mainly due to its dependence on a predefined list of sensitive APIs, which can quickly become outdated as Android versions evolve, potentially overlooking critical behaviors. Mamadroid performs the worst, with an accuracy of only 66.30% and a precision of just 60.40%. The main reason lies in its abstraction of API calls into package families, which leads to the loss of significant semantic information, thereby reducing classification performance.

Overall, the experimental results indicate that the proposed model effectively leverages information from multiple components of Android applications by extracting and combining behavioral and permission features. Additionally, the stacking-based ensemble learning architecture plays a key role in enhancing classification performance by integrating the strengths of various machine learning algorithms.

#### 4.4.6. Experimental results of scenario 6.

This scenario evaluates the cross-dataset generalization capability of HybFusion on the Drebin benchmark dataset. HybFusion is compared with baseline methods, including DexRay [14], NATICUSdroid [21], Permission [28], Malscan [24], Mamadroid [48], and S^3^Feature [7]. All models were trained on the primary experimental dataset described in Section 4.1 and then tested on unseen Drebin malware samples. Since Drebin contains only malware samples, we report the True Positive Rate (TPR), which measures the proportion of malware correctly detected, and the False Negative Rate (FNR), which measures the proportion of malware incorrectly classified as benign. Detailed results are presented in Table 10.

**Table 10:** Cross-dataset detection performance on Drebin (%).

Method	TPR	FNR
DexRay [14]	94.12	5.88
NATICUSdroid [21]	96.85	3.15
Permission [28]	96.02	3.98
Malscan [24]	97.11	2.89
Mamadroid [48]	93.02	6.98
S^3^Feature [7].	73.35	26.65
HybFusion	**98.15**	**1.85**

The results in Table 10 demonstrate that HybFusion achieves the best cross-dataset detection performance among all compared methods, obtaining the highest TPR of 98.15% and the lowest FNR of 1.85%. These results indicate that the proposed model can effectively detect previously unseen malware samples from a different dataset.

Among the baseline methods, Malscan achieves the best baseline performance with a TPR of 97.11% and an FNR of 2.89%. Nevertheless, HybFusion still surpasses Malscan by improving the TPR by 1.04% while further reducing the FNR to 1.85%. This performance gain suggests that combining behavioral and semantic permission features yields more robust representations.

Overall, the results confirm that HybFusion exhibits strong cross-dataset generalization capability and maintains high detection effectiveness on unseen malware samples.

#### 4.4.7. Experimental results of scenario 7.

To evaluate the robustness of HybFusion under evasion attacks, adversarial experiments were conducted using four common attack strategies: Permission stuffing, FCG poisoning, Name obfuscation, and Permission reordering. These attacks simulate realistic manipulation techniques that preserve application functionality while attempting to evade detection.

(1) Permission stuffing: The attacker injects benign permissions into the *AndroidManifest.xml* file to dilute malicious semantic signals. In this experiment, 100 benign permissions were added to each malicious sample.
(2) FCG poisoning: The attacker inflates the FCG with dummy benign nodes and edges to perturb graph aggregation in the GNN. In this experiment, call indirection transformations were applied using the Obfuscapk [49] tool to insert additional intermediate method calls without altering program semantics.
(3) Name Obfuscation: The attacker renames original class and method identifiers with meaningless symbols to remove semantic information embedded in class and method names. This transformation was implemented using the Obfuscapk tool.
(4) Permission reordering: The attacker shuffles the order of permissions declared in the *AndroidManifest.xml* file to evaluate permutation sensitivity in Transformer-based embeddings. In this experiment, permissions were randomly shuffled 100 times per sample to assess prediction stability under permutation.

We use clean accuracy and adversarial accuracy to evaluate the detection performance under clean and adversarial conditions. In addition, the Attack Success Rate (ASR) is adopted to measure the effectiveness of each attack. The ASR is defined as:


$$\begin{array}{c} ASR=\frac{N_{flip}}{N_{correct}}, \end{array}$$ (7)

where $N_{correct}$ denotes the number of malware samples correctly classified under clean conditions, and $N_{flip}$ denotes the number of such samples that become misclassified after the attack.

Table 11 presents the robustness evaluation results of HybFusion under four representative evasion attack strategies. The experimental results indicate that HybFusion maintains stable detection performance under adversarial manipulations, with adversarial accuracy remaining above 97.8% across all evaluated scenarios.

**Table 11:** Robustness evaluation of HybFusion under adversarial attacks (%).

Attack Type	Clean Accuracy	Adversarial Accuracy	ASR
Permission stuffing	99.27	98.77	1.01
FCG poisoning	99.27	98.11	2.34
Name obfuscation	99.27	97.83	2.91
Permission reordering	99.27	98.98	0.60

Under the permission stuffing attack, where 100 benign permissions are injected to dilute malicious semantic signals, the model achieves an adversarial accuracy of 98.77% with an ASR of 1.01%. This result suggests that the feature fusion strategy mitigates the impact of semantic dilution, as behavioral features extracted from the FCG complement the permission-based representation.

For the permission reordering attack, the model achieves the highest adversarial accuracy of 98.98% and the lowest ASR of 0.60% among the evaluated attacks. This result suggests that the permission-based semantic representation remains relatively stable under permutation.

In the case of FCG poisoning, adversarial accuracy decreases slightly to 98.11% with an ASR of 2.34%. Despite the structural perturbations resulting from call indirection, the model maintains strong detection performance, suggesting that the GNN captures robust structural patterns within the call graph.

Regarding name obfuscation, the adversarial accuracy is 97.83% with an ASR of 2.91%, indicating a slightly larger impact compared with the other attacks. Even when the semantic information embedded in class and method names is removed, HybFusion maintains strong performance by leveraging structural topology features such as in-degree, out-degree, closeness centrality, Katz centrality, and clustering coefficient.

Overall, the experimental results demonstrate that HybFusion maintains strong robustness against multiple evasion strategies. Across all evaluated attacks, the adversarial accuracy remains above 97.8% while the ASR stays below 3%, indicating that the proposed model preserves stable detection capability under realistic adversarial manipulations.

#### 4.4.8. Experimental results of scenario 8.

This scenario evaluates the resilience of HybFusion against several common malware obfuscation techniques, including manifest randomization, class and method renaming, call indirection, and constant string encryption. We use the Obfuscapk tool to generate obfuscated malware samples from the original test set. The experimental results are summarized in Table 12.

**Table 12:** Performance of HybFusion under various obfuscation techniques (%).

Obfuscation Setting	Accuracy	Precision	Recall	F1-score
Original (Non-obfuscated)	99.27	99.30	99.24	99.27
Manifest randomization	98.78	99.29	98.26	98.78
Class and Method Renaming	97.83	99.28	96.36	97.80
Call indirection	98.11	99.28	96.92	98.09
Constant string encryption	98.26	99.28	97.23	98.24

The results in Table 12 show that HybFusion maintains strong detection performance across common malware obfuscation techniques, with F1-score remaining above 97.80%.

Class and method renaming leads to the most noticeable performance reduction, with the F1-score decreasing to 97.80%. This result is expected because identifier renaming removes semantic information embedded in function and class names. Nevertheless, the model still maintains stable detection performance by leveraging the structural role of nodes in the FCG and integrating permission-based semantic features.

The remaining obfuscation techniques have only a limited impact on the model. Specifically, call indirection yields an F1-score of 98.09%, indicating that the GIN-based graph representation learning remains robust against malware that obfuscates the structure of the FCG. Constant string encryption results in an F1-score of 98.24%, suggesting that the feature representation does not heavily rely on string literals. In contrast, manifest randomization causes only a minor performance change, with an F1-score of 98.78%.

#### 4.4.9. Experimental results of scenario 9.

This scenario evaluates the computational efficiency of HybFusion by analyzing its runtime performance and resource consumption. All experiments were conducted on the Kaggle platform equipped with an Intel Xeon 2.00 GHz CPU, 13 GB of RAM, and an NVIDIA Tesla P100 GPU with 16 GB of VRAM.

Table 13 summarizes the computational cost and throughput of the proposed model. The results show that HybFusion processes each application with an average runtime of 5.5 seconds per sample. This processing speed is particularly notable considering that the underlying FCGs may contain thousands of nodes, while the experiments were conducted in a relatively modest computing environment. With an observed throughput of approximately 655 applications per hour, HybFusion demonstrates strong potential for scalability. In parallel processing environments, the analysis capacity can be further increased. Moreover, deploying the system on mid-range or high-performance computing platforms may further improve the processing speed.

**Table 13:** Computational cost and throughput analysis of HybFusion.

Metric	Value
Average processing time per sample	5.5 s
Peak RAM usage	50.47 MB
Peak VRAM usage	96.65 MB
Throughput	655 apps/hour

In addition to runtime efficiency, HybFusion exhibits low memory consumption, with peak RAM and VRAM usage of 50.47 MB and 96.65 MB, respectively. Notably, the GPU memory consumption accounts for less than 1% of the available 16 GB VRAM, indicating that the model requires only modest computational resources.

These results highlight the practical deployability and scalability of HybFusion, making it well suited for large-scale Android malware analysis scenarios where thousands of applications must be processed efficiently.

### 4.5. Discussion

#### 4.5.1. Advantages of the proposed model.

The experimental results presented in this study demonstrate the superior performance of the proposed model compared to other approaches. We believe there are three main reasons contributing to the model’s high performance.

a) **Effective feature extraction and fusion strategy**

The results from Scenario 1 indicate that combining behavioral and permission features significantly enhances malware detection performance compared to using a single feature type independently. Specifically, the model employing combined features achieves an F1-score of 99.27%, whereas using only behavioral or permission features individually yields 98.57% and 97.49%, respectively. This confirms that the two feature types are complementary, enabling the model to comprehensively capture malicious behaviors.

Regarding the behavioral feature extraction method, our approach relies on a function call graph with enriched nodes. This rich representation, when combined with GIN, which is one of the powerful GNN architectures, enables the model to effectively learn complex behavioral patterns of malware.

For the permission feature extraction method, this study is among the first to apply the pre-trained language model all-MiniLM-L6-v2 to embed permission lists into a semantic representation. This approach allows the model to learn semantic relationships between permissions, thereby improving its ability to distinguish between normal permissions and those potentially abused by malware.

In summary, each feature extraction method effectively captures a distinct aspect of the application. By integrating these complementary features, the model is able to construct a more comprehensive behavioral profile, which significantly improves malware detection performance compared to approaches that rely on a single feature type.

b) **Effectiveness in semantic feature extraction from permission lists**

The results from Scenario 2 demonstrate that the proposed permission feature extraction method using the pre-trained all-MiniLM-L6-v2 language model outperforms traditional approaches. This advantage stems from the capability of Transformer-based models to capture latent semantic relationships among normalized permissions. Unlike discrete representations or traditional embedding techniques like Word2vec, the all-MiniLM-L6-v2 model enables global representation learning for permission sequences, thereby providing richer semantic features for the classifier.

c) **Effectiveness of stacking ensemble learning technique**

The results from Scenario 3 show that the classification model using the proposed stacking-based ensemble learning architecture significantly outperforms individual classifiers such as KNN, MLP, RF, and XGBoost. Specifically, the ensemble model achieves the highest performance across all metrics, with an accuracy of 99.27%, precision of 99.30%, recall of 99.24%, and F1-score of 99.27%. This demonstrates that combining multiple machine learning algorithms can effectively leverage the strengths of individual base classifiers, thereby enhancing the overall performance of the model.

#### 4.5.2. Threats to validity.

Although the experimental results demonstrate that the proposed method achieves high malware detection performance, this study still has some limitations.

First, the large size and complex structure of the function call graph can significantly increase computational costs and affect the effectiveness of graph representation learning. In this study, the entire FCG is used as input to the GNN model; however, not all nodes and edges reflect malicious behaviors. Without applying graph optimization techniques, such as pruning less relevant components, the input representation may contain noise, which reduces the quality of learned features. This not only affects the model’s performance but also limits its scalability.

Second, the model relies solely on static features, while modern malware variants often employ sophisticated evasion techniques such as code obfuscation, packing, or dynamic code loading to bypass detection mechanisms. These behaviors are typically difficult to capture through static analysis and only manifest during runtime. As a result, the lack of dynamic features may reduce the model’s ability to detect malware, especially in cases involving complex obfuscation techniques.

Third, although the all-MiniLM-L6-v2 model enables effective semantic representation learning for permission features, it has an inherent limitation in input length, with a maximum context size of 256 tokens. In a small number of cases where applications declare unusually long permission lists, truncation of the input sequence may lead to partial information loss, particularly if sensitive permissions appear toward the end of the list.

Fourth, the potential impact of concept drift represents a significant challenge in the evolving Android malware landscape. Although our dataset spans multiple years, the rapid evolution of malware may affect long-term detection performance. In addition, potential dataset bias may influence the model’s ability to generalise to previously unseen malware families, as the dataset may not fully capture the diversity of real-world malware ecosystems. While the hybrid feature representation in HybFusion is designed to capture robust behavioral patterns, the model’s effectiveness against future or previously unseen malware variants requires further validation through continuous temporal evaluation.

Fifth, the stacking-based ensemble introduces additional computational overhead compared to individual classifiers. Specifically, the training time increases from 47.88 seconds for the best-performing individual classifier (RF) to 111.13 seconds for the stacking model, corresponding to an increase of approximately 2.3 times. In addition, the average inference time per sample at the classification stage increases from 0.02 seconds to 0.07 seconds. This reflects the additional computational cost required to achieve improved detection performance.

## 5. Conclusion

This paper proposes HybFusion, a novel approach for Android malware detection that effectively leverages two key types of features: behavioral features extracted using graph neural networks and permission features obtained using pre-trained language models applied to normalized permission sequences. By integrating these features into a stacking-based ensemble learning model, HybFusion significantly enhances detection performance. Experimental results show that HybFusion outperforms existing methods across all evaluation metrics, achieving an F1-score of 99.27%. Future work can focus on the following directions. First, function call graphs extracted from Android applications are often large and complex. Therefore, developing effective graph optimization techniques, such as pruning unnecessary nodes or edges, can help reduce computational cost while still keeping the core behavioral information. This may improve the representational ability and feature learning efficiency of GNNs. Second, we suggest that incorporating dynamic features in a well-designed manner could be an effective way to enhance detection performance, especially for malware that uses advanced evasion techniques. Third, future work may investigate more robust permission modeling strategies to alleviate the input length limitation of Transformer-based encoders.
